# Heart Sound Classification with MFCCs and Wavelet Daubechies Analysis Using Machine Learning Algorithms

**DOI:** 10.3390/diagnostics16010083

**Published:** 2025-12-26

**Authors:** Sebastian Guzman-Alfaro, Karen E. Villagrana-Bañuelos, Manuel A. Soto-Murillo, Jorge Isaac Galván-Tejada, Antonio Baltazar-Raigosa, Angel Garcia-Duran, José María Celaya-Padilla, Andrea Acuña-Correa

**Affiliations:** 1 Unidad Académica de Ingeniería Eléctrica, Universidad Autónoma de Zacatecas, Jardín Juárez 147, Centro, Zacatecas 98000, Mexico; 2 Unidad Académica de Medicina Humana, Universidad Autónoma de Zacatecas, Jardín Juárez 147, Centro, Zacatecas 98000, Mexico

**Keywords:** heart sounds, heart diseases, machine learning, MFCC, wavelet analysis, computer-aided diagnosis

## Abstract

**Background/Objectives:** Cardiovascular diseases are the leading cause of mortality worldwide according to the World Health Organization (WHO), highlighting the need for accessible tools for early detection. Automated classification systems based on signal processing and machine learning offer a non-invasive alternative to support clinical diagnosis. **Methods:** This study implements and evaluates machine learning models for distinguishing normal and abnormal heart sounds using a hybrid feature extraction approach. Recordings labeled as normal, murmur, and extrasystolic were obtained from the PASCAL dataset and subsequently binarized into two classes. Multiple numerical datasets were generated through statistical features derived from Mel-Frequency Cepstral Coefficients (MFCCs) and Daubechies wavelet analysis. Each dataset was standardized and used to train four classifiers: support vector machines, logistic regression, random forests, and decision trees. **Results:** Model performance was assessed using accuracy, precision, recall, specificity, F1-score, and area under curve. All classifiers achieved notable results; however, the support vector machine model trained with 26 MFCCs and Daubechies-4 wavelet coefficients obtained the best performance. **Conclusions:** These findings demonstrate that the proposed hybrid MFCC–Wavelet framework provides competitive diagnostic accuracy and represents a lightweight, interpretable, and computationally efficient solution for computer-aided auscultation and early cardiovascular screening.

## 1. Introduction

Cardiovascular diseases are the leading cause of death worldwide and in Mexico, according to the National Institute of Public Health and the National Institute of Statistics and Geography (INEGI) [1,2]. In 2024, 144,912 deaths were registered in Mexico, representing 23.74% of all deaths in the country during that period. It is estimated that approximately 20.5 million people die from heart disease annually worldwide, emphasizing the urgent need for accessible and early diagnostic tools. Conventional auscultation, although widely used, relies heavily on clinician experience and subjective interpretation [3]. In this regard, the automation of heart sound (phonocardiogram, PCG) analysis through artificial intelligence (AI) has become an active area of research. However, PCG signals are non-stationary, brief, and often contaminated by noise from respiration, motion, or external environments, which complicates their analysis.

Developing tools capable of not only monitoring the cardiac cycle but also making autonomous decisions to generate accurate diagnoses could improve healthcare by helping address physician shortages, such as the shortage of specialized physicians [4].

Cardiac auscultation is performed using a stethoscope, the most commonly used medical instrument in cardiology [5,6,7]. This type of cardiac monitoring technique offers several advantages because, from an engineering perspective, it enables the extraction of key information to detect subtle cardiac abnormalities that may not be audible to clinicians [8].

Computer-assisted analysis (CAD) has given rise to computer-assisted cardiac auscultation (CAA), which is an artificial intelligence system integrated into a stethoscope. Together, these systems have preprocessing capabilities that include filtering and enhancing the signal emitted by the heart, segmentation to detect important cardiac events, classification to predict whether the captured sound is pathological or not, and finally, evaluation to measure the performance of the integrated system [9,10].

Recent research efforts have focused on extracting handcrafted and learned features from PCG signals to discriminate between normal and pathological heart sounds. Traditional approaches have relied on time–frequency analysis, wavelet decomposition, and cepstral-based descriptors to capture morphological and spectral variations between cardiac cycles [11,12,13,14]. More recent studies have investigated hybrid models that combine wavelet subband features with neural networks, demonstrating enhanced representational power for biomedical signals [15]. For instance, hybrid Wavelet–ANN architectures have been successfully applied in physiological signal analysis, confirming the complementarity between wavelet-based decomposition and neural network learning [16].

In parallel, time-growing or dynamic neural networks have emerged as a promising class of models capable of adapting their topology as new data arrive, providing greater flexibility for handling temporal and non-stationary biomedical signals [17]. Such architectures have been reported in cardiac signal research to capture evolving patterns across heart cycles. Deep learning approaches, particularly CNN-based frameworks, have also shown strong potential in heart sound classification, achieving state-of-the-art accuracy on benchmark datasets [18,19]. Nonetheless, these models often require large training datasets and substantial computational resources, which limit their applicability in low-resource and embedded settings.

Several studies have explored heart sound classification using machine learning techniques. For example, Yunendah Nur Faudah et al. [20] employed support vector machines (SVM) and random forest (RF) using the PhysioNet 2016 database [21], working with Mel-Frequency Cepstral Coefficients and achieving accuracy scores of 0.77 and 0.91. Similarly, Jinhui Li et al. [22] applied the SVM classifier to the same PhysioNet 2016 dataset [21], obtaining accuracy values of up to 0.79.

In this context, recent studies have shown that the combination of Mel-Frequency Cepstral Coefficients (MFCCs) and wavelet-derived statistical features provides a robust representation of heart sound signals due to their ability to capture complementary spectral and temporal patterns. MFCCs emulate the perceptual behavior of the human auditory system and have demonstrated strong discriminative capabilities in cardiac murmur classification tasks, particularly when used with traditional machine learning algorithms [23]. Wavelet transforms, on the other hand, offer a multiresolution analysis well suited for the non-stationary and transient nature of phonocardiogram recordings, enabling the decomposition of S1, S2, and murmur components across several frequency bands [24]. Recent machine learning frameworks integrating MFCC and wavelet coefficients have reported improved performance in distinguishing normal from pathological heart sounds by leveraging the complementary strengths of both domains [25,26]. Building upon these findings, the present study proposes a practical, computationally efficient, and interpretable pipeline that integrates handcrafted MFCC- and Daubechies-based features for binary heart sound classification. Unlike previous works, this approach explicitly implements subject-level data splitting and nested cross-validation to prevent information leakage, ensuring unbiased and reproducible results. The proposed framework seeks to remain interpretable and computationally efficient, and enable future implementation on embedded or low-resource diagnostic devices suitable for rural clinical environments.

## 2. Main Contributions

Although the individual components employed in this study—such as MFCCs, wavelet-based descriptors, and traditional machine learning classifiers—have been previously explored in the literature, this work provides a structured and reproducible framework that integrates these elements under rigorous experimental conditions. The main contributions of this study can be summarized as follows:

1. A leakage-free and reproducible experimental pipeline for heart sound classification is presented, incorporating subject-level data splitting and nested cross-validation to prevent information leakage, which is often overlooked in prior studies using small biomedical datasets.
2. A systematic evaluation of hybrid MFCC–Wavelet feature representations is conducted across multiple configurations, analyzing the impact of different MFCC dimensionalities and Daubechies wavelet families on classification performance.
3. An interpretable and computationally efficient classification framework is proposed, demonstrating that compact handcrafted feature sets (as few as eight features) can achieve competitive diagnostic performance, making the approach suitable for real-time and embedded auscultation systems.
4. A comprehensive comparison of feature selection strategies (Forward Selection and Boruta) is provided, highlighting their influence on dimensionality reduction, model stability, and diagnostic metrics relevant to clinical decision support.

Collectively, these contributions emphasize practical deployment, interpretability, and methodological rigor, offering a viable alternative to data-intensive deep learning approaches in resource-limited clinical environments.

## 3. Methods and Experimentation

**Figure 1:**
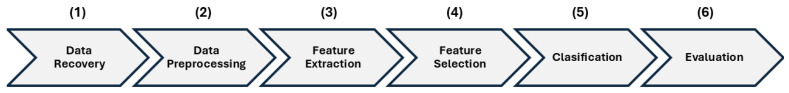
Overview of the methodological workflow for heart sound classification. The process is divided into six stages: (1) Data Recovery, obtained from the PASCAL heart sound dataset; (2) Data Preprocessing, including normalization and duration adjustment; (3) Feature Extraction, using Mel-Frequency Cepstral Coefficients (MFCCs) and Wavelet–Daubechies analysis; (4) Feature Selection, applying Forward and Boruta methods; (5) Classification, through supervised machine learning algorithms; and (6) Evaluation, based on performance metrics to identify the best-performing model.

The overall methodology employed in this study is summarized in Figure 1, which provides the complete flowchart of the proposed classification model. The diagram outlines the six sequential stages of the workflow and illustrates how the outputs of each block serve as inputs to the next, enabling a clear visualization of the end-to-end processing pipeline. In  Data Recovery (1), the recordings were obtained from the Classifying Heart Sounds Challenge (PASCAL) database, which includes labeled samples of normal, murmur, and extrasystolic sounds. Data Preprocessing (2) involved adjusting all recordings to a fixed duration of three seconds and normalizing their amplitude to ensure uniformity and approximate common clinical recording conditions. During Feature Extraction (3), two complementary digital signal processing techniques were applied—Mel-Frequency Cepstral Coefficients (MFCCs) and Wavelet–Daubechies analysis—to capture spectral and temporal information. Feature Selection (4) consisted of identifying the most informative statistical descriptors to reduce dimensionality and improve the classifier’s discriminative capacity. In Classification (5), four supervised machine learning algorithms—Logistic Regression (LR), Support Vector Machines (SVM), Random Forests (RF), and Decision Trees (DT)—were implemented. Finally, Evaluation (6) consisted of assessing model performance using Accuracy, Precision, Recall (Sensitivity), Specificity, F1-Score, and Area Under the Curve (AUC), selecting the configuration that achieved the best overall results.

### 3.1. Database Acquisition

The dataset employed in this study was obtained from the Classifying Heart Sounds Challenge (PASCAL) [27]. This publicly available database was recorded in a real clinical environment using a digital stethoscope (Digiscope!). It comprises a total of 312 heart sounds samples recordings in “.wav” format, collected from patients with different cardiac conditions. The distribution of the recordings across the corresponding classes is summarized in Table 1.

Clinically, normal heart sounds are characterized by the fundamental sound pattern (S1 and S2) produced by closing the atrioventricular and semilunar valves, respectively, and generally have a regular rhythm without additional acoustic events [28]. In contrast, extrasystolic sounds arise from premature cardiac contractions that momentarily disrupt the normal cardiac cycle, producing an additional beat often perceived as an irregularity or pause [29].

Heart murmurs, on the other hand, originate from turbulent blood flow within the heart or great vessels and can be classified according to their intensity, duration, and position within the cardiac cycle. Although murmurs are not always pathological—since they may also occur under physiological conditions such as increased cardiac output or in pediatric patients—for the purposes of this study, and consistent with the annotations provided in the PASCAL database, all murmur recordings were classified as abnormal. This categorization ensures a clear separation between normal and pathological phonocardiogram (PCG) patterns, facilitating subsequent analysis and classification [30].

### 3.2. Dataset Pre-Processing

The classes were binarized into normal sounds and abnormal sounds, with the latter including both murmur and extrasystolic recordings. This decision was made because the extrasystolic class internally contains other pathological patterns, such as gallop rhythms or clicks [5,31]. Likewise, although heart murmurs are not always indicative of disease since they can also occur under benign physiological conditions, such as increased cardiac output or in pediatric patients, they were classified as abnormal in this study. This decision is consistent with the annotations provided in the PASCAL database, where murmurs were labeled as pathological due to their association with altered hemodynamic conditions or valvular dysfunctions. Therefore, both extrasystolic and murmur sounds were grouped under the abnormal category. The resulting class redistribution is presented in Table 2.

To simulate the most realistic possible clinical environment during data processing, no audio data augmentation techniques such as those reported in the literature involving the addition of Gaussian noise [32] were applied. Instead, a temporal trimming procedure was implemented, considering that the physiological average duration of a cardiac cycle is approximately 0.8 s [33]. Given that certain cardiac pathologies cannot be identified within a single cycle, it was essential to ensure that each audio segment contained more than one complete cardiac cycle. Therefore, a fixed duration of three seconds per recording was established, corresponding to approximately four cardiac cycles.

**Figure 2:**
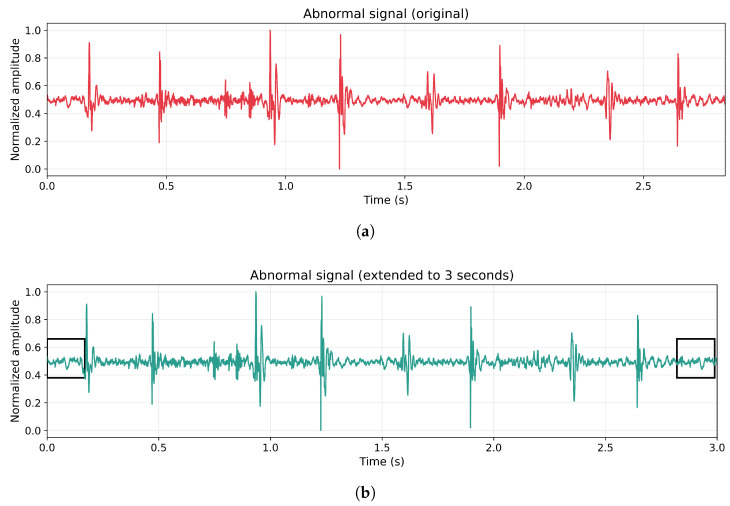
(**a**) Abnormal cardiac signal not meeting the time threshold. (**b**) Temporally extended abnormal cardiac signal adjusted to a duration of 3 s. The black boxes indicate the original signal segments reused to extend the recording length. In this example, it was not necessary to append the full 0.8-s cardiac cycle; only the required portion was added, extracted from the initial segment of the recording to reach the established threshold.

In the case of normal heart sound recordings, meeting this time criterion did not represent a problem. However, twenty abnormal recordings did not satisfy the minimum duration and were subjected to a temporal extension procedure, as illustrated in Figure 2. This extension consisted of duplicating the signal segment corresponding to one cardiac cycle (0.8 s) and appending it to the end of the original recording until the total duration reached three seconds. This approach avoided the use of artificially synthesized data, preserving the natural characteristics of the signal while maintaining its cyclic behavior. Additionally, all abnormal recordings were retained for subsequent analysis, ensuring that no abnormal samples were excluded.

Finally, from the 200 normal recordings, 112 samples were randomly selected in order to obtain fully balanced classes resulting in the final dataset used for the classification models, as shown in Table 3.

Before describing the implementation details of the feature extraction pipeline, this section first presents the theoretical background of the Mel-Frequency Cepstral Coefficient (MFCC) method and the Wavelet–Daubechies transform. These techniques form the basis of the proposed hybrid representation and justify the extraction of perceptually and physiologically relevant descriptors from heart sound recordings. After introducing the theoretical foundations, the subsequent subsections detail the specific parameter configurations and processing steps used in this study.

### 3.3. Feature Extraction

Heart sounds signal recordings are non-stationary biomedical signals composed of transient acoustic events associated with the mechanical activity of the heart. The primary components, S1 and S2, present distinct temporal and spectral patterns, while pathological murmurs exhibit broader frequency content and variable duration. For this reason, feature extraction methods must capture both time-localized characteristics and frequency-dependent structure.

Mel-Frequency Cepstral Coefficients (MFCCs) and Wavelet–Daubechies analysis were employed with the objective of developing a hybrid classification model that combines the perceptual advantages of MFCCs with the multiresolution capabilities of wavelet analysis. This dual approach was designed to exploit both the spectral and temporal information contained in heart sound signals, thereby enhancing the discriminative power of the resulting features.

#### 3.3.1. Mel-Frequency Cepstral Coefficients (MFCC)

**Figure 3:**
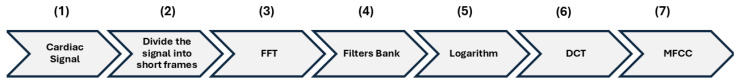
Diagram of the MFCC extraction process. The procedure comprises seven stages: (1) acquisition of the cardiac signal, (2) division of the signal into short overlapping frames, (3) computation of the Fast Fourier Transform (FFT), (4) application of Mel-scale filter banks, (5) logarithmic compression of spectral energies, (6) Discrete Cosine Transform (DCT), and (7) generation of the final Mel-Frequency Cepstral Coefficients (MFCCs).

MFCCs constitute one of the most widely used techniques in audio analysis, particularly in speech and biomedical signal processing. They are based on the emulation of the human auditory system, which operates through a series of perceptual filters whose amplitude magnitude decays exponentially and whose center frequencies are logarithmically spaced. MFCCs approximate this behavior by applying filter banks on the Mel scale to determine perceptually relevant spectral components [34,35]. Figure 3 illustrates the main stages involved in the MFCC extraction process.

Following the flow in Figure 3, the first and second stage involves dividing the cardiac signal (1) into temporal frames (2) to capture as much information as possible from the signal. In this process, a smoothing function must be applied to avoid amplitude drops at the edges of the frames, which could otherwise introduce unwanted noise into the analysis (Equation (1));

(1) x[n]=w[n]s[n],

where x[n] denotes the windowed signal, w[n] the smoothing function, and s[n] the original signal.

In this study, each 3-s recording was segmented into 25-ms frames (as this duration effectively captures the fundamental heart sounds), with a 10-ms step size, ensuring an overlap of 60%. This configuration balances temporal resolution and redundancy, yielding approximately 298 frames per audio segment. The Bartlett (triangular) window was adopted as the smoothing function due to its low computational cost and its capability to attenuate border effects (Equation (2));

(2) $$w\left(n\right)=\left\{\begin{array}{cc} \frac{2n}{N}, & 0\leq n\leq \frac{N}{2},\\ 2-\frac{2n}{N}, & \frac{N}{2}\leq n\leq N, \end{array}\right.$$

where *N* represents the window length.

Subsequently, each frame was transformed from the time domain to the frequency domain using the Fast Fourier Transform (FFT) (3), as defined in Equation (3) [36];

(3) $$X\left[k\right]=\sum_{n=0}^{N-1}x\left[n\right]\cdot e^{-j\frac{2\pi kn}{N}},\;k=0,1,\ldots ,N-1.$$

The resulting magnitude spectrum was processed using a set of Mel filter banks (4) to approximate the nonlinear frequency response of the human auditory system. The mapping between the linear frequency scale and the Mel scale is expressed in Equation (4) [37];

(4) $$m=2595\cdot \log_{10}\;\left(1+\frac{f}{700}\right),$$

where *f* denotes the frequency in Hertz and *m* the corresponding frequency in the Mel scale. This transformation expands low-frequency regions and compresses high-frequency regions, providing greater perceptual resolution at lower frequencies. The triangular Mel filters are mathematically defined in Equation (5);   

(5) $$B\left(m,k\right)=\left\{\begin{array}{cc} 0, & k<f(m-1),\\ \frac{k-f(m-1)}{f(m)-f(m-1)}, & f(m-1)\leq k\leq f(m),\\ \frac{f(m+1)-k}{f(m+1)-f(m)}, & f(m)\leq k\leq f(m+1),\\ 0, & k>f(m+1), \end{array}\right.$$

where B(m,k) is the filter bank matrix, *m* is the filter bank index, and *k* is the number of analyzed frames.

Before applying the logarithmic compression, the energy for each band is computed as shown in Equation (6) [38];

(6) $$E_{m}=\sum_{f}{\left|X(f)\right|}^{2}\cdot H_{m}\left(f\right),$$

where ${\left|X(f)\right|}^{2}$ represents the power spectral density of the signal, $H_{m}\left(f\right)$ is the *m*-th filter response, and $E_{m}$ corresponds to the total energy captured by that filter. Twenty Mel filters were used, yielding a vector of 20 energies corresponding to distinct spectral regions.

A logarithmic transformation (5) is then applied to approximate the nonlinear perception of intensity in the auditory cortex (Equation (7));

(7) $$E_{\log}=\log\left(E_{m}\right)$$

To minimize correlation among features and compact the relevant information, the Discrete Cosine Transform (DCT) (6) is subsequently applied, producing the Mel-Frequency Cepstral Coefficients (MFCCs) (7) (Equation (8));

(8) $$MFCC\left(n\right)=\sum_{m=1}^{M}E_{\log}\left(m,k\right)\cos\left[n\left(m-\frac{1}{2}\right)\frac{\pi }{M}\right],\;m=1,2,\ldots ,M,$$

where *m* is the number of MFCC coefficients, which varies with respect to n=1,2,...,M. Here *n* represents the number of filter banks, and *k* the number of frames for the analysis.

For this study, 8, 12, 16, 20 and 26 MFCCs were extracted to generate independent datasets, later integrated with wavelet-based features. To manage dimensionality, two statistical descriptors—mean and standard deviation—were computed per coefficient, resulting in feature vectors of 16, 24, 32, 40, and 52 dimensions, respectively.

#### 3.3.2. Wavelet–Daubechies Analysis

Wavelets are particularly suitable for the analysis of non-stationary biomedical signals such as phonocardiograms. They enable multiresolution decomposition of a signal into components with distinct temporal and spectral resolutions. Mathematically, the general form of a wavelet function is given in Equation (9) [39];

(9) $$\psi_{a,b}\left(t\right)=\frac{1}{\sqrt{\left|a\right|}}\;\psi \;\left(\frac{t-b}{a}\right)\;a\neq 0,$$

where *t* denotes time, *b* the translation parameter (temporal shift), and *a* the dilation or scale factor. The Discrete Wavelet Transform (DWT) was adopted in this study due to its orthogonality, efficiency, and suitability for non-stationary signal analysis [40]. For a discrete signal x(t), the DWT is expressed as (Equation (10));

(10) $$W_{j,k}=\sum_{i=0}^{n-1}x_{i}\;2^{j/2}\;\psi *\;\left(2^{j}t_{i}-k\right),$$

where *j* and *k* correspond to the scale and translation indices, respectively, and  $\psi^{*}\left(\cdot \right)$ denotes the complex conjugate wavelet function.

The DWT decomposes the signal through iterative low-pass (approximations, A) and high-pass (details, D) filtering, generating sub-bands at successive resolutions. Four levels of decomposition were applied, yielding sub-bands D1–D4 and A4. Given a sampling frequency of $f_{s}=4000$ Hz, the corresponding frequency ranges are summarized in Table 4.

The Daubechies wavelet family was selected for its compact support, orthogonality, and proven efficacy in heart sound analysis. Specifically, Daubechies-4, Daubechies-6, and Daubechies-8 were used to generate distinct feature sets. From each sub-band (D1–D4 and A4), six statistical descriptors were computed: mean, standard deviation, variance, energy, Shannon energy, and Shannon entropy. Consequently, each wavelet-based dataset comprised 30 features, ensuring a consistent representation across decomposition levels.

After the independent extraction processes, the feature vectors obtained from the MFCC and Wavelet–Daubechies analyses were integrated into a single hybrid representation. The fusion was performed by concatenating z-score normalized feature vectors from both domains, ensuring that all variables contributed equally to the learning process and preventing dominance by features with larger numerical ranges. This hybrid representation combines perceptual and spectral–temporal information, improving model generalization.

Regarding the selection of statistical descriptors, each measure was chosen to capture distinct physiological and signal characteristics. The mean and standard deviation quantify the amplitude distribution and variability across frames, while the variance emphasizes local energy fluctuations. The energy and Shannon energy provide insight into the signal’s power concentration across sub-bands—key indicators of heart sound intensity and abnormal cardiac activity. Finally, the Shannon entropy measures the complexity and irregularity of the signal, which increases in pathological phonocardiograms. These statistics are well established in biomedical signal processing for their interpretability, low computational complexity, and physiological relevance [38,39,40].

As a result, the final hybrid dataset integrates complementary features from both MFCC and Wavelet–Daubechies analyses, capturing perceptual, temporal, and spectral patterns essential for robust and generalizable heart sound classification.

### 3.4. Feature Selection

Feature selection plays a fundamental role in machine learning applications, as it contributes to the construction of simpler, more interpretable, and less overfitted models. In this study, two complementary feature selection methods were applied: Forward Selection and Boruta. Both techniques were implemented exclusively on the training set, ensuring that no information from the test data influenced the selection process, thereby preventing data leakage. All procedures were implemented in Python.

The Forward Selection method was implemented using the SequentialFeatureSelect function from scikit-learn. This approach iteratively evaluates subsets of features by starting from an empty set and progressively adding the variable that produces the greatest improvement in model performance according to the F1-macro score [41]. A 5-fold cross-validation scheme was applied within the training set to assess each subset’s performance. To determine the optimal number of features, different configurations were tested in increments of five, ranging from 5 to 25 features. The final subset was selected based on the configuration that yielded the highest performance according to the corresponding classifier.

In parallel, the Boruta algorithm was applied as a wrapper method around a random forest classifier. Boruta identifies all features that are statistically relevant by comparing the importance of real features against randomly permuted “shadow” features, introducing controlled randomness to obtain a robust and unbiased ranking [42]. This method was implemented using the BorutaPy package, configured with 100 base estimators and automatic adjustment of the number of trees.

The feature subsets obtained from both selection strategies were subsequently used to train and evaluate the classification models. For comparative purposes, the performance of each feature selection method was primarily assessed using Accuracy, F1-score, and the Area Under the ROC Curve (AUC), which provide a balanced overview of both global and class-wise performance. These results were later complemented by additional evaluation metrics—such as Precision, Recall, and Specificity—during the final model assessment to ensure a comprehensive performance analysis.

### 3.5. Classification

Through the feature extraction process, a total of 15 datasets were generated. Before being used in the classification stage, all datasets were subjected to Z-score standardization (Equation (11)) to normalize the feature scales, ensuring that each variable had a mean of zero and unit variance. This preprocessing step prevents features with larger numerical ranges from dominating the model training process;

(11) $$X_{\mathrm{stand}}=\frac{X-\mu }{\sigma },$$

where $X_{\mathrm{stand}}$ is the standardized value, *X* is the original value, μ is the mean of the original value, and σ represents the standard deviation.

In this study, four supervised machine learning algorithms were implemented in Python using the scikit-learn library: Logistic Regression (LR), Support Vector Machine (SVM), Decision Tree (DT), and Random Forest (RF). These models were selected for their interpretability, stability, and computational efficiency, making them suitable for datasets of limited size such as the one used in this study (224 samples). Although deep learning models such as Convolutional Neural Networks (CNNs) have achieved remarkable performance in larger datasets, they generally require extensive data augmentation and significant computational resources, which may lead to overfitting when applied to smaller, handcrafted-feature datasets. Therefore, traditional classifiers were preferred to maintain interpretability and ensure reproducibility under realistic clinical and embedded conditions.

Each classifier was optimized using GridSearchCV with 5-fold cross-validation, allowing an exhaustive hyperparameter search while avoiding information leakage. The configuration details of each algorithm are summarized as follows:

- Support Vector Machine (SVM): The SVM algorithm aims to find an optimal decision boundary, or hyperplane, that effectively separates data samples belonging to different classes while minimizing overfitting, making it well-suited for binary classification problems [43]. In this study, the SVM was configured with a radial basis function (RBF) kernel. The regularization parameter *C* and kernel coefficient γ were optimized within the ranges C∈[0.1,1,10,100] and γ∈[scale,0.01,0.1,1]. This configuration allows the model to maximize the margin between normal and abnormal heart sound classes while minimizing classification errors.
- Logistic Regression (LR): Analyzes the relationship between multiple independent variables and a categorical dependent variable, estimating the probability of an event by fitting the data to a logistic curve [44]. In this study, the model was implemented in Python using the scikit-learn.linear_model module within a pipeline that included feature standardization through StandardScaler. The configuration employed the *lbfgs* optimization solver with L2 regularization, a maximum of 1000 iterations, and a fixed random seed of 42 to ensure reproducibility. Additionally, hyperparameter optimization was performed using GridSearchCV with a 5-fold cross-validation scheme applied exclusively to the training data, preventing information leakage and ensuring reliable hyperparameter tuning. This setup provided a stable and interpretable baseline classifier capable of estimating the probability of normal and abnormal heart sound classes while maintaining low computational complexity.
- Decision Tree (DT): Decision Trees model the relationship between input features and possible outcomes by simulating a hierarchical tree structure based on the concept of information gain (entropy) [45]. In this study, the DT classifier was configured using multiple splitting criteria (*gini*, *entropy*, and *log-loss*), with maximum depths ranging from 3 to 12 and minimum samples per leaf between 1 and 6. The algorithm recursively partitions the feature space to identify thresholds that maximize class separability while minimizing node impurity. Hyperparameter optimization was conducted using GridSearchCV with a 5-fold cross-validation scheme applied only to the training data, ensuring a balance between complexity and generalization.
- Random Forest (RF): Random Forest is an ensemble-based machine learning approach that integrates multiple decision trees through bootstrap aggregation (bagging) and random feature selection to improve classification robustness [46]. In this study, the RF classifier was configured using an ensemble of trees trained on different bootstrap samples of the data, where each tree was built from a random subset of features at each split. The optimized hyperparameters included the number of trees ($n_{\mathrm{estimators}}\;=\;200,500,1000$), maximum depth (5–12), and feature sampling strategies (*sqrt*, *log*2, and 0.5). This ensemble method enhances generalization by averaging the predictions of multiple decision trees, effectively reducing variance and mitigating overfitting while maintaining high predictive accuracy.

All experiments were conducted on a personal workstation equipped with an 11th Gen Intel^®^ Core™ i7-11800H processor (2.30 GHz), Intel Corporation, Santa Clara, CA, USA), 16 GB of RAM (15.7 GB usable), and Windows 11 (64-bit), using Python 3.11 and the *scikit-learn* library (version 1.5). GPU acceleration was not required due to the low computational cost of the models. Training time varied depending on the number of selected features and the classifier used: the Random Forest (RF) model exhibited the longest training time (approximately four minutes), whereas the Logistic Regression (LR), Support Vector Machine (SVM), and Decision Tree (DT) models completed training within a few seconds. This efficiency indicates that the system can be deployed in real-time or embedded computer-aided auscultation applications.

For each of the 15 datasets, two classification models were trained—one using Forward-selected features and another using Boruta-selected features—employing an 80/20 stratified split for training and testing, respectively. The resulting models were subsequently evaluated using multiple performance metrics to assess their diagnostic reliability.

### 3.6. Evaluation Metrics

To evaluate model performance, several metrics were computed using sklearn.metrics, matplotlib, seaborn and numpy libraries. Both numerical and graphical analyses were performed to assess the classification results comprehensively [47]. The metrics are defined as follows:

- Accuracy: This metric provides the percentage of individuals that are correctly classified (Equation (12)).
  (12) $$Accuracy=\frac{True\;Positives+True\;Negatives}{Total\;Examples}$$
- Recall (Sensitivity or True Positive Rate): Measures the proportion of positive cases that were correctly detected by the model (Equation (13)).
  (13) $$Recall=\frac{True\;Positives}{True\;Positives+False\;Negatives}$$
- Specificity (True Negative Rate): Measures the proportion of actual negatives that are correctly identified as such (Equation (14)).
  (14) $$Specificity=\frac{True\;Negatives}{False\;Positives+True\;Negatives}$$
- Precision: Measures the consistency of results when measurements are repeated. (Equation (15)).
  (15) $$Precision=\frac{True\;Positives}{False\;Positives+True\;Positives}$$
- F1-score: It is the harmonic mean of Precision and Recall. (Equation (16)).
  (16) $$F1-Score=2\cdot \frac{Precision\cdot Recall}{Precision+Recall}$$
- ROC (Receiver Operating Characteristic Curve): Provides an overall measure of diagnostic accuracy, independent of the decision threshold and prevalence. It is obtained by plotting sensitivity (or recall) on the vertical axis and 1-specificity on the horizontal axis. From this metric, the Area Under the Curve (AUC) is derived, ranging from 0 to 1.

## 4. Results

The dimensional characteristics of the feature vectors generated during the MFCC and Wavelet–Daubechies extraction stages, as well as the number of descriptors retained by the Advanced Selection and Boruta algorithms, are shown below. These elements summarize the outcome of each processing block for constructing the hybrid feature sets used for classification. Table 5 detailing the number of MFCC coefficients, the type of Daubechies wavelet, and the total number of features generated in each configuration.

Table 5 lists the 15 hybrid datasets generated. The first column enumerates the dataset identifier, the second reports the number of extracted MFCC coefficients, the third specifies the Daubechies wavelet family employed (with four levels of decomposition), and the fourth indicates the total number of combined features. Two classification models were trained for each feature selection method (Forward and Boruta), resulting in eight classifiers per dataset and a total of 120 models. Among these, the 29 best-performing configurations were selected for detailed analysis, followed by the identification of the six most outstanding models (highlighted in bold in Table 6).

Table 6 is organized into nine columns. The first column lists the classification technique used, while the second specifies the feature selection approach and the number of selected features. The remaining columns summarize the evaluation metrics used for comparison, including Accuracy, Precision, Recall (Sensitivity), Specificity, $F_{1}$-score, and Area Under the Curve (AUC).

In contrast, the hybrid MFCC–Wavelet approach presented in this study, combined with Forward Selection or Boruta, yielded improved overall performance, as reflected in the highest achieved accuracy of 0.7556 and an AUC of up to 0.8419. This comparison highlights the contribution of each processing block and supports the effectiveness of the proposed multi-stage architecture relative to established baseline methodologies.

Based on the obtained results, the highest accuracy value reached was 0.7556, achieved by eleven models employing SVM, LR, and DT techniques across different datasets. This result indicates good overall performance for distinguishing between normal and abnormal heart sounds.

Regarding sensitivity (recall), the highest value recorded was 0.8636, obtained exclusively by the SVM model using Boruta with eight selected features on dataset 13. This metric reflects the model’s ability to correctly identify pathological cases, which is particularly relevant in clinical practice, as a higher sensitivity reduces the likelihood of false negatives—that is, failing to detect a patient with an actual cardiac abnormality.

In contrast, the highest specificity value was 0.9565, achieved by decision tree classifiers with Boruta 7 on datasets 9, 12, and 15. This denotes a strong capability to recognize normal cardiac sounds, minimizing false positives. Nevertheless, these models exhibited lower sensitivity, which implies that some pathological cases could go undetected—a limitation that must be carefully considered in diagnostic applications where missing abnormal events is clinically critical.

For the precision metric, the best result obtained was 0.9231, also produced by the same decision tree classifiers. This demonstrates the model’s robustness in correctly classifying abnormal samples without mislabeling normal ones. Regarding the $F_{1}$-score, the highest value achieved was 0.8235 using the logistic regression model with Boruta 8 on dataset 13, representing a balanced trade-off between sensitivity and precision.

Finally, for the AUC metric, the maximum value of 0.8419 was achieved by three models employing SVM and LR techniques. This value reflects the models’ strong discriminative ability across varying decision thresholds, which is desirable for automated cardiac screening tools. From a clinical standpoint, these results suggest that the proposed models can serve as a reliable decision-support mechanism for identifying abnormal heart sounds, especially in resource-limited environments or rural healthcare settings where access to specialized diagnostic equipment is restricted.

**Figure 4:**
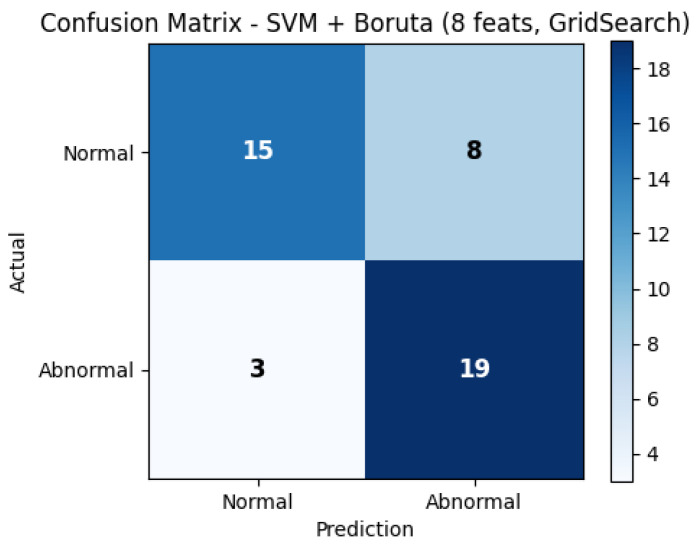
Confusion matrix of the best-performing SVM model (Boruta 8, dataset 13). The matrix shows a balanced classification performance with high sensitivity for abnormal heart sounds (true positives) and acceptable specificity for normal sounds, confirming the model’s practical reliability.

**Figure 5:**
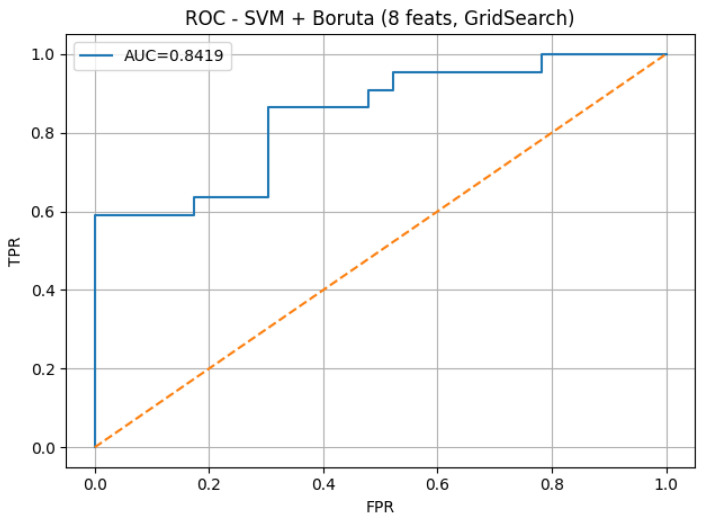
Receiver Operating Characteristic (ROC) curve of the best-performing model (SVM with Boruta 8 on dataset 13). The model achieved an AUC of 0.8419, demonstrating strong discriminative capability between normal and abnormal heart sounds across different decision thresholds. The dashed diagonal line represents the performance of a random classifier (AUC = 0.5), serving as a baseline for comparison.

To further illustrate the diagnostic performance of the best-performing model, Figure 4 presents the confusion matrix, and Figure 5 shows the corresponding ROC curve. Both plots correspond to the SVM model with Boruta feature selection using 8 features on dataset 13, which achieved the highest sensitivity (0.8636) and an AUC of 0.8419.

Figure 5 presents the ROC curve of the best-performing model, corresponding to the SVM classifier trained with Boruta-selected features on dataset 13. The curve shows an AUC value of 0.8419, which indicates a strong discriminative power between normal and abnormal heart sounds. This value reflects the model’s robustness in differentiating pathological cardiac events under varying classification thresholds.

Complementarily, Figure 4 illustrates the confusion matrix for the same model. A balanced distribution of true positives and true negatives can be observed, confirming that the classifier achieves adequate sensitivity for detecting abnormal sounds while maintaining acceptable specificity for normal cases. These findings reinforce the potential of the proposed hybrid MFCC–Wavelet approach as a reliable computational tool for supporting clinical auscultation.

## 5. Discussion

To contextualize the diagnostic performance of the proposed methodology, the findings of this study were compared with previous research on heart sound classification employing MFCCs, wavelet-based descriptors, and traditional machine learning models. This comparative analysis allows assessing the effectiveness of the hybrid MFCC–Wavelet representation relative to single-domain feature extraction approaches reported in the literature. Furthermore, the discussion integrates results from studies using both traditional algorithms and more recent deep learning architectures, showing how the proposed pipeline compares with current methods.

To evaluate the effectiveness of the proposed method, the results were compared with baseline approaches reported in recent studies employing MFCCs, wavelet-based features, or traditional machine learning models for heart sound classification [23,24,25,26]. These works commonly rely on single-domain feature representations and do not incorporate hybrid spectral–temporal descriptors or feature selection mechanisms optimized at the dataset level.

According to the results obtained from Table 6, the best-performing model was the SVM classifier using Boruta-selected features (dataset 13). When analyzing the most relevant metrics for computer-aided diagnosis (CAD)—namely accuracy, AUC, and recall—it becomes evident that each offers a complementary perspective on diagnostic performance. Sensitivity (recall) measures the system’s ability to correctly identify abnormal cardiac sounds, a critical parameter in clinical applications where false negatives (missed pathological cases) can lead to severe consequences. Accuracy represents the overall rate of correctly classified instances, and in this study, given that the dataset was fully balanced, it serves as an objective indicator of performance. Finally, the AUC provides an aggregate measure of discriminative ability across all possible decision thresholds, quantifying how well the system distinguishes between normal and pathological heart sounds.

When compared with previous works using the same PASCAL dataset, the present results demonstrate competitive or superior performance with a more compact feature set. For instance, Zeilani and Akhavan [48] reported an accuracy of 81% using Random Forests and 75% using SVM, based on a combination of statistical, frequency-based, and information-theoretic features. Similarly, Soto-Murillo [49] achieved 73.17% accuracy using logistic regression trained on 52 handcrafted features that included temporal, spectral, and linear predictive coding descriptors. In contrast, the proposed hybrid MFCC–Wavelet model achieved an accuracy of 75.56% and an AUC of 0.8419 while relying on only 8 features selected by the Boruta algorithm. this reduction in dimensionality reduces model complexity but also facilitates real-time applicability in embedded and portable systems.

In recent years, other studies have explored more complex architectures for heart sound classification. For example, Kalatehjari et al. [18] and Wang et al. [19] implemented deep convolutional neural networks (CNNs) that achieved accuracies above 95% using large datasets with thousands of samples. However, these models require high computational resources, GPU acceleration, and extensive data preprocessing, which limits their deployment in low-resource clinical environments. By contrast, the present study emphasizes interpretability and efficiency—two essential factors for real-time applications such as digital stethoscopes or telemedicine devices.

Recent studies have also proposed specialized validation schemes for adaptive and time-growing neural architectures, such as the A-Test validation framework described by Gharehbaghi [50], while this approach is well suited for incremental deep learning models that evolve over time, it is not directly applicable to static classifiers trained on handcrafted features, as used in the present study. Nevertheless, such validation strategies highlight important directions for future work when extending heart sound analysis toward adaptive or deep learning-based frameworks.

Beyond individual deep learning studies, recent survey works have systematically reviewed the use of MFCC-based representations for heart sound classification. In particular, a comprehensive review on deep learning methods for heart sound signal analysis reports classification accuracies reaching up to 100% when MFCC features are combined with high-capacity neural architectures, while these results demonstrate the strong representational potential of MFCCs within deep learning frameworks, such performance is typically achieved under idealized conditions, including large-scale datasets, extensive data augmentation strategies, and complex network architectures. In contrast, the present study focuses on a lightweight and interpretable pipeline, employing strict leakage-free validation, limited data availability, and traditional machine learning classifiers. Under these realistic and constrained conditions, the proposed hybrid MFCC–Wavelet approach achieves competitive diagnostic performance while prioritizing transparency, reproducibility, and feasibility for embedded or resource-limited clinical applications [51].

In addition, recent developments in neural architectures for biomedical signal processing have explored more complex temporal–frequency representations compared with handcrafted MFCC–Wavelet features. For instance, time-growing neural networks have demonstrated excellent performance in detecting short-duration cardiac events such as the systolic ejection click, achieving high sensitivity (98.1%) and strong robustness to noise by progressively expanding temporal windows to capture evolving spectral cues [52]. Similarly, recent work on spiking neural networks (SNNs) has shown that neuromorphic hardware and event-driven computation can process biosignals such as EEG and ECG with markedly lower energy consumption, real-time inference, and accuracy comparable to that of deep learning models, positioning SNNs as promising candidates for on-device medical AI [53]. In parallel, CNN-based approaches that transform 1D signals into 2D representations using methods such as Continuous Wavelet Transform (CWT), Short-Time Fourier Transform (STFT), or Recurrence Plots (RPs) have achieved near-perfect accuracy in ECG and EEG classification tasks by leveraging richer time–frequency structures [54]. Compared with these architectures, the method proposed in this study prioritizes interpretability, low computational cost, and suitability for small datasets—key requirements for real-time auscultation systems and portable diagnostic tools, while TGNNs, SNNs, and 2D CNNs may outperform traditional classifiers under large and well-curated datasets, they typically require higher computational resources, substantial training corpora, and less interpretability. Thus, the hybrid MFCC–Wavelet framework presented here offers a practical and explainable alternative for resource-limited environments, maintaining competitive diagnostic performance without the complexity of modern deep architectures.

MFCCs present several intrinsic limitations when applied to biomedical signals: they were originally developed for speech processing, their logarithmic compression can mask diagnostically relevant amplitude variations, and the Discrete Cosine Transform may remove temporal structure that carries clinical significance in heart sounds. Furthermore, MFCCs are sensitive to noise and recording artifacts, which can distort the spectral envelope and affect downstream classification. In light of these limitations, the hybrid approach used in this study helps counterbalance the weaknesses of MFCCs by incorporating wavelet-based features, which preserve transient components and time–frequency details that MFCCs alone may fail to capture. This integration yields a more stable and meaningful representation, particularly important when working with short or noisy heart sounds recordings.

A critical limitation of this study is the relatively small dataset size (224 balanced recordings), which constrains the generalization of the model. The dataset also includes heterogeneous recording conditions that introduce acoustic variability and potential redundancy between samples. Furthermore, heart sounds exhibit intrinsic non-stationarity—particularly in the temporal positioning and intensity of S1 and S2 events—making segmentation a challenging task. Although the fixed 3-s window ensures consistency across samples, this approach may omit fine-grained timing variations between cardiac cycles. Future work could address this limitation by incorporating adaptive segmentation or dynamic time warping (DTW) to align cardiac events temporally.

From a clinical perspective, the proposed approach offers several advantages in terms of interpretability. Unlike deep learning models, which operate as black boxes, the hybrid MFCC–Wavelet representation provides transparent, physiologically meaningful features. The MFCCs capture the spectral envelope of heart sounds, analogous to how physicians perceive pitch and tone during auscultation, while the wavelet subbands characterize transient events corresponding to the S1 and S2 phases. This alignment between computational and perceptual domains enhances the clinical trustworthiness of the system, facilitating its integration into decision-support tools. Moreover, the compact feature space allows the model to be implemented in embedded microcontrollers, enabling the development of portable diagnostic systems for rural or resource-limited healthcare settings.

Overall, the results demonstrate that traditional machine learning classifiers, when combined with well-designed handcrafted features and robust feature selection, can achieve competitive diagnostic accuracy compared to deep neural networks. The hybrid MFCC–Wavelet framework thus provides a practical balance between interpretability, computational efficiency, and diagnostic performance, reinforcing its potential as a foundation for real-time, explainable cardiac auscultation systems.

## 6. Conclusions

The present work introduces a hybrid approach that balances diagnostic performance, interpretability, and computational efficiency. The proposed model integrates Mel-Frequency Cepstral Coefficients (MFCCs) with wavelet-based statistical features, enabling the extraction of both spectral and temporal characteristics of phonocardiogram (PCG) signals.

The best configuration—an SVM classifier trained with eight Boruta-selected features—achieved an accuracy of 75.56%, an AUC of 0.8419, and a recall of 0.8636. These results demonstrate that a compact and interpretable feature set can reach competitive diagnostic performance compared to more complex deep learning approaches, while maintaining strong sensitivity for detecting abnormal cardiac events.

This methodology offers advantages in terms of simplicity, low computational cost, and suitability for embedded implementations, making it a a suitable approach for real-time or portable systems. However, the limited dataset size and variability in phonocardiogram recordings indicate that larger datasets are needed for validation and the exploration of adaptive segmentation methods. Future work may also extend this framework toward multiclass classification to differentiate specific cardiac pathologies such as murmurs, gallops, or arrhythmias.

## Figures and Tables

**Table 1:** Distribution of recordings in the PASCAL heart sound dataset.

Category	Number of Recordings
Normal	200
Murmurs	66
Extrasystolic	46
Total recordings	312

**Table 2:** Binarized distribution of heart sound recordings from the PASCAL dataset. Murmur and extrasystolic categories were grouped as abnormal sounds based on their clinical association with pathological cardiac events.

Category	Number of Recordings
Normal	200
Abnormal	112
Total recordings	312

**Table 3:** Binarized and balanced Dataset Categories.

Category	Number of Recordings
Normal	112
Abnormal	112
Total recordings	224

**Table 4:** Frequency ranges covered by detail (D) and approximation (A) coefficients.

Level	Detail (D) Covers Frequencies (Hz)	Approximation (A) Covers Frequencies (Hz)
D1	1000–2000	0–1000
D2	500–1000	0–500
D3	250–500	0–250
D4	125–250	0–125
A4	0–125	

**Table 5:** Summary of the hybrid datasets generated for classification. Each dataset combines MFCC coefficients with wavelet-based features extracted using Daubechies functions at four decomposition levels.

Dataset	MFCC Features	Wavelet Type	Total Features
1	8	Db4	46
2	8	Db6	46
3	8	Db8	46
4	12	Db4	54
5	12	Db6	54
6	12	Db8	54
7	16	Db4	62
8	16	Db6	62
9	16	Db8	62
10	20	Db4	70
11	20	Db6	70
12	20	Db8	70
13	26	Db4	82
14	26	Db6	82
15	26	Db8	82

**Table 6:** Performance metrics of the 29 best-performing classification models across different hybrid feature sets.

Model	Selector	Number of Features	Dataset (Table 5)	Accuracy	Precision	Recall	Specificity	$F_{1}$-Score	AUC ^1^
SVM ^2^	Boruta	9	7	0.6222	0.6000	0.6818	0.5662	0.6383	0.8004
SVM	Boruta	7	8	0.7368	0.7111	0.6364	0.7826	0.6829	0.8100
SVM	Boruta	8	10	0.7556	0.7037	0.8336	0.6522	0.7755	0.8419
SVM	Forward	10	5	0.7111	0.6800	0.7727	0.6522	0.7234	0.8261
SVM	Boruta	6	6	0.6667	0.6670	0.6364	0.6957	0.6512	0.8053
SVM	Boruta	7	13	0.7556	0.7037	0.8636	0.6522	0.7755	0.8419
SVM	Forward	25	14	0.7556	0.7200	0.8182	0.6957	0.7666	0.7885
SVM	Boruta	7	14	0.6667	0.6400	0.7273	0.6087	0.6809	0.8251
LR	Boruta	6	1	0.7333	0.7500	0.6816	0.7826	0.7143	0.8399
LR	Boruta	7	4	0.7333	0.7500	0.6818	0.7826	0.7143	0.8419
LR ^3^	Forward	20	2	0.7111	0.7368	0.6364	0.7526	0.6829	0.8182
LR	Boruta	8	10	0.7556	0.8235	0.6364	0.8996	0.7179	0.8360
LR	Boruta	7	4	0.7333	0.7500	0.6818	0.7826	0.7143	0.8419
LR	Forward	10	5	0.7111	0.6800	0.7727	0.6522	0.7334	0.8300
LR	Forward	25	13	0.7556	0.7391	0.7725	0.7391	0.7556	0.7806
LR	Boruta	8	13	0.7556	0.8235	0.6364	0.8996	0.8235	0.8360
LR	Forward	25	14	0.7556	0.7619	0.7273	0.7826	0.7442	0.8063
LR	Boruta	7	14	0.7111	0.7368	0.6364	0.7826	0.6829	0.8103
RF ^4^	Boruta	6	3	0.6889	0.7000	0.6364	0.7391	0.6667	0.8261
RF	Boruta	7	6	0.6889	0.7000	0.6364	0.7391	0.6667	0.8261
RF	Forward	10	7	0.6889	0.7000	0.6364	0.7391	0.6667	0.8083
RF	Boruta	7	9	0.7111	0.7143	0.6818	0.7391	0.6977	0.8142
RF	Boruta	7	12	0.7111	0.7143	0.6818	0.7391	0.6877	0.8142
RF	Boruta	7	15	0.7111	0.7143	0.6818	0.7391	0.6877	0.8142
DT	Boruta	6	3	0.6889	0.7000	0.6364	0.7391	0.6667	0.8261
DT ^5^	Boruta	7	6	0.7556	0.8235	0.6364	0.8696	0.7179	0.8103
DT	Boruta	7	9	0.7556	0.9231	0.5455	0.9565	0.6857	0.8271
DT	Boruta	7	12	0.7556	0.9231	0.5455	0.9565	0.6857	0.8271
DT	Boruta	7	15	0.7556	0.9231	0.5455	0.9565	0.6857	0.8271
SVM	Boruta	8	13	0.7556	0.7037	0.8636	0.6522	0.7755	0.8419

^1^ Area Under the Curve, ^2^ Support Vector Machine, ^3^ Logistic Regression, ^4^ Random Forest, ^5^ Decision Tree.

## Data Availability

Publicly available datasets were analyzed in this study. This data can be found here: peterjbentley.com/heartchallenge/taskoverview (accessed on 1 September 2024).
